# Selection of reference genes for miRNA qRT-PCR under abiotic stress in grapevine

**DOI:** 10.1038/s41598-018-22743-6

**Published:** 2018-03-13

**Authors:** Meng Luo, Zhen Gao, Hui Li, Qin Li, Caixi Zhang, Wenping Xu, Shiren Song, Chao Ma, Shiping Wang

**Affiliations:** 1 Department of Plant Science, School of Agriculture and Biology, Shanghai Jiao Tong University, Shanghai, 200240 China; 2 Center for Viticulture and Enology, School of Agriculture and Biology, Shanghai Jiao Tong University, Shanghai, 200240 China; 3 Institute of Agro-food Science and Technology, Key Laboratory of Agro-products Processing Technology of Shandong, Shandong Academy of Agricultural Sciences, Jinan, 250100 China

## Abstract

Grapevine is among the fruit crops with high economic value, and because of the economic losses caused by abiotic stresses, the stress resistance of *Vitis vinifera* has become an increasingly important research area. Among the mechanisms responding to environmental stresses, the role of miRNA has received much attention recently. qRT-PCR is a powerful method for miRNA quantitation, but the accuracy of the method strongly depends on the appropriate reference genes. To determine the most suitable reference genes for grapevine miRNA qRT-PCR, 15 genes were chosen as candidate reference genes. After eliminating 6 candidate reference genes with unsatisfactory amplification efficiency, the expression stability of the remaining candidate reference genes under salinity, cold and drought was analysed using four algorithms, geNorm, NormFinder, deltaCt and Bestkeeper. The results indicated that *U6 snRNA* was the most suitable reference gene under salinity and cold stresses; whereas *miR168* was the best for drought stress. The best reference gene sets for salinity, cold and drought stresses were *miR160e* + *miR164a*, *miR160e* + *miR168* and *ACT* + *UBQ* + *GAPDH*, respectively. The selected reference genes or gene sets were verified using *miR319* or *miR408* as the target gene.

## Introduction

Grapevine is a fruit crop with high economic value that is widely cultivated worldwide^1^. Nevertheless, grapevine is sensitive to climate change, which often causes a great loss in grape or wine production when unfavourable climate occurs in both vegetative and reproductive growth periods^2,3^. Salinity, cold and drought are major abiotic stresses in grapevine and in other plants^4,5^. With the global climate change that has occurred in recent years, the adverse effects caused by these abiotic stresses may be aggravated in the foreseeable future^6^. Environmental stressors can damage cellular structure and may lead to the physiological function disorders^7^, which are manifested in impaired membranes, dysfunctional proteins and increased reactive oxygen species (ROS)^8,9^. As a result, salinity, cold and drought stress can strongly influence the yield and quality of grapevine and sometimes, can even be lethal for grapevine^8^. Therefore, salinity, cold and drought resistance has always received focused attention of researchers. Epigenetic regulation, such as methylated modification and post-transcriptional regulation, is the stress response mechanism of plants, which can affect the resistance to stresses^10,11^. Among the post-transcriptional regulators, microRNAs (miRNAs) are likely of great importance^12^. The miRNAs are a class of small, non-coding RNAs with a length of 20–24 nucleotides^13^. In plants, miRNAs are produced from pri-miRNAs with hairpin structure, which are processed by a protein complex containing DICER-LIKE1 (DCL1), SERRATE (SE) and HYL1^14,15^. miRNAs regulate the expression of the target gene by cleavage or translational repression^14^. In the complex gene regulatory network of plants, miRNAs are key regulatory factors that normally influence the expression of downstream genes by regulating their transcription factors^16^. In this way, miRNAs participate in many physiological activities, such as development, flowering and stress resistance^17^. Further understanding of the stress-related molecular mechanisms of miRNAs will provide new prospects in grapevine resistance breeding through molecular and genetic engineering methods^18^.

Currently, various methods of miRNA identification and quantification have been developed, such as northern blot^19^, microarray^20^ and high-throughput sequencing^21^. As a widely used method for gene expression analysis, the qRT-PCR (quantitative reverse transcriptase-polymerase chain reaction) is applied to identify protein-coding gene abundance in many plants. However, using qRT-PCR to detect miRNA abundance due to its small size was a great challenge until stem-loop^22^ and poly (A)-tailed^23^ qRT-PCR approaches were developed to characterize mature miRNA abundance. Because of the sensitivity, accuracy and large dynamic range, qRT-PCR soon became the most commonly used technique for miRNA quantification^24,25^. Although qRT-PCR is a powerful tool for gene expression analysis, normalization is required in the analysis system, because the quantification results can be easily influenced by a few factors, such as the RNA quality, design of reverse transcription primer and the efficiency of reverse transcription enzyme^26^. Reference genes are widely used as an internal control in qRT-PCR because the expression of the reference gene can be regarded stable under different experimental conditions and in different tissues. To guarantee the reliability of normalization, the use of multiple reference genes is suggested by Vandesompele *et al*.^27^ Notable, the reference gene suitable for all experimental conditions does not exist; thus, the selection of appropriate reference genes for qRT-PCR in different plant species or different stress conditions is necessary. In previous studies, various reference genes have been used in plant species. Among these genes, 5.8S ribosomal RNA (*5.8S rRNA*) and U6 small nuclear RNA (*U6 snRNA*) are the most commonly used^28,29^. However, the potential of miRNAs as reference genes has received close attention recently. As reported by Feng *et al*.^30^, the expression of miRNAs is more stable than that of protein-coding genes under biotic and abiotic stresses. Research related to the selection of reference genes for miRNA qRT-PCR has been performed in wheat^30^, soybean^31^, lettuce^32^, peach^33^ and sugarcane^28^. Nevertheless, until now, no report on miRNA reference gene evaluation in grapevine has been available. Therefore, suitable reference genes for grapevine miRNA study are urgently required, particularly for use in stress resistance research.

In this study, the experiment was designed to select the most suitable reference genes for grapevine miRNA qRT-PCR assays under abiotic stresses (salinity, cold, and drought stress). Fifteen candidate reference genes, including 4 traditional housekeeping genes, Actin (*ACT*, GenBank Accession: EC969944), Ubiquitin (*UBQ*, GenBank Accession: EC929411), Glyceraldehyde 3-phosphate dehydrogenase (*GAPDH*, GenBank Accession: AT1G13440.1), and Elongation factor1-alpha (*EF1*, GenBank Accession: AT5G60390.1), 9 miRNAs (*miR156a, 159a, 160e, 162, 164a, 167a, 168, 169a*, and *396a*), and 2 non-coding reference genes, *5.8S rRNA* (GenBank Accession: KT344661.1) and *U6 snRNA* (Location: chr6_15577690_15577792_+), were chosen for expression stability evaluation by the stem-loop qRT-PCR method. The amplification characteristics of these candidate reference genes were assessed first, and then, the Cq values of candidates with acceptable amplification efficiency and specificity were used to evaluate the expression stability using the algorithms of geNorm^27^, NormFinder^34^, Bestkeeper^35^ and deltaCt^36^. Based on the results obtained, the suitable reference genes in salinity, cold, drought stresses were provided, which promoted a more accurate miRNA qRT-PCR assay.

## Results

### Amplification characteristic and Cq range of candidate reference genes

The candidate reference genes consisted of 2 non-coding RNAs, 4 housekeeping genes and 9 miRNAs. The sequences and stem-loop primers of these selected miRNAs are shown in Table 1. To confirm the efficient amplification of pair-primers, the standard curves were obtained using a set of diluted cDNA templates. Based on the criteria of standard curve^27^, the regression coefficient (R^2^) should be more than 0.98 with the amplification efficiency (E) tending to 100%. According to the selecting criteria, *miR156a*, *miR159a*, *miR162*, *miR167a*, *miR169a* and *miR396a* were excluded from the candidates because of an unsatisfactory E value or R^2^ (Table 2). Among the remaining candidate reference genes, the E values ranged from 1.97 to 2.17, and R^2^ varied between 0.982 and 0.999. Moreover, the single melting peak during real-time PCR verified the amplification specificity of the final 9 candidate reference genes (Fig. S1).

**Table 1:** The sequences and stem-loop primers of candidate reference miRNAs.

Gene	Sequence	Stem-loop primer (5′-3′)
miR156a	TGACAGAAGAGAGGGAGCAC	GTCGTATCCAGTGCAGGGTCCGAGGTATTCGCACTGGATACGACGTGCTCCC
miR159a	CTTGGAGTGAAGGGAGCTCTC	GTCGTATCCAGTGCAGGGTCCGAGGTATTCGCACTGGATACGACGAGAGCTC
miR160e	TGCCTGGCTCCCTGTATGCCA	GTCGTATCCAGTGCAGGGTCCGAGGTATTCGCACTGGATACGACTGGCATAC
miR162	TCGATAAACCTCTGCATCCAG	GTCGTATCCAGTGCAGGGTCCGAGGTATTCGCACTGGATACGACCTGGATGC
miR164a	TGGAGAAGCAGGGCACGTGCA	GTCGTATCCAGTGCAGGGTCCGAGGTATTCGCACTGGATACGACTGCACGTG
miR167a	TGAAGCTGCCAGCATGATCTG	GTCGTATCCAGTGCAGGGTCCGAGGTATTCGCACTGGATACGACCAGATCAT
miR168	TCGCTTGGTGCAGGTCGGGAA	GTCGTATCCAGTGCAGGGTCCGAGGTATTCGCACTGGATACGACTTCCCGAC
miR169a	CAGCCAAGGATGACTTGCCGG	GTCGTATCCAGTGCAGGGTCCGAGGTATTCGCACTGGATACGACCCGGCAG
miR396a	TTCCACAGCTTTCTTGAACTA	GTCGTATCCAGTGCAGGGTCCGAGGTATTCGCACTGGATACGACTAGTTCAA

**Table 2:** The primers and amplification characteristics of candidate reference genes.

Gene	Gene code	Forward primer (5′-3′)	Reverse primer (5′-3′)	Efficiency	R^2^
5.8S rRNA	KT344661.1	GCTCTCGCATCGATGAAGAAC	AGACTCGATGATTCACGGGATT	2.03	0.997
U6 snRNA	u6wg_chr6_15577690_15577792_+	CCGATAAAATTGGAACGATACAGAG	TCGATTTGTGCGTGTCATCCT	2.13	0.995
ACT	EC969944	GACAATGGATGGACCAGATTCA	CTTGCATCCCTCAGCACCTT	1.99	0.997
UBQ	EC929411	GTGGGTGCAGACGTGCATAA	CCTTGATGCAATTGGCTAGGA	2.14	0.991
GAPDH	CB973647.1	AGCCCTCAACGAGAAGTTCTTG	TCGATCACACGGGAGCTGTA	1.97	0.995
EF1	EC959059	AACCGAAAGCACCTCGATCA	TGGTTGAGTCCTTTGCTTTTCC	1.98	0.999
miR160e	MIMAT0005656	GCGGCGGTGCCTGGCTCC	GTGCAGGGTCCGAGGT	2.10	0.982
miR164a	MIMAT0005658	GCGGCGGTGGAGAAGCAG	GTGCAGGGTCCGAGGT	2.05	0.997
miR168	MIMAT0005675	GCGGCGGTCGCTTGGTGC	GTGCAGGGTCCGAGGT	2.17	0.996
miR156a	MIMAT0005640	GCGGCGGTGACAGAAGAG	GTGCAGGGTCCGAGGT	2.09	0.977
miR159a	MIMAT0005648	GCGGCGGCTTGGAGTGAA	GTGCAGGGTCCGAGGT	2.37	0.976
miR162	MIMAT0005657	GCGGCGGTCGATAAACCT	GTGCAGGGTCCGAGGT	2.57	0.989
miR167a	MIMAT0005670	GCGGCGGTGAAGCTGCCA	GTGCAGGGTCCGAGGT	3.31	0.911
miR169a	MIMAT0005676	GCGGCGGCAGCCAAGGAT	GTGCAGGGTCCGAGGT	2.34	0.987
miR396a	MIMAT0005724	GCGGCGGTTCCACAGCTT	GTGCAGGGTCCGAGGT	2.46	0.958

**Figure 1:**
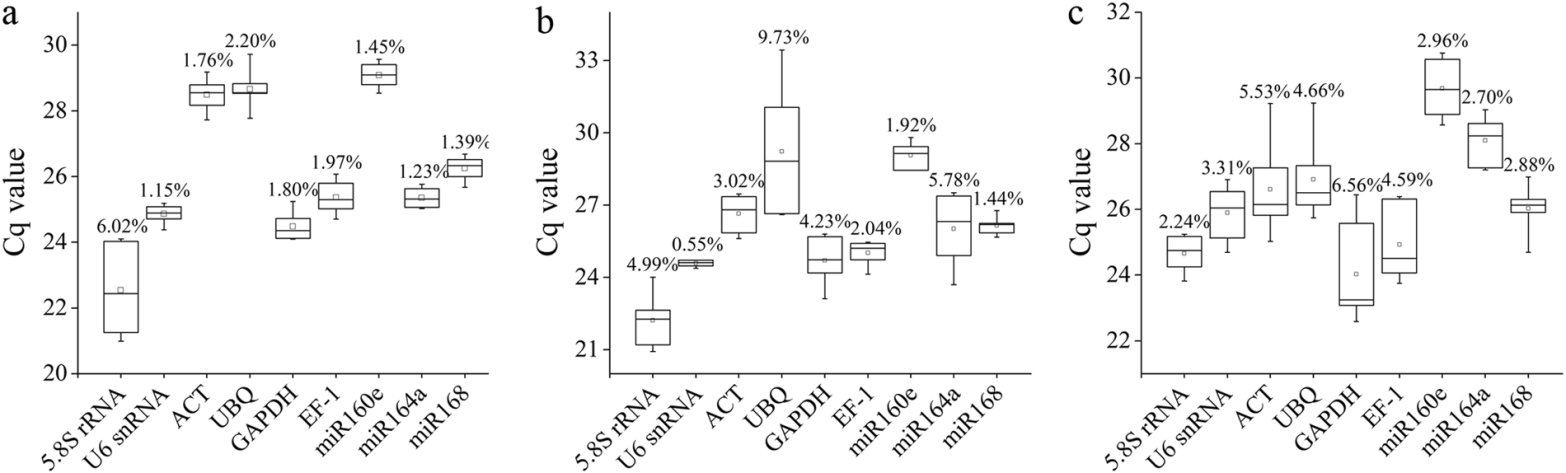
The expression levels of candidate reference genes. The Cq values generated by qRT-PCR of six time points under different abiotic stresses are shown as box plots. Whisker caps, boxes, lines and square boxes represent maximum/ minimum, 25/75 percentiles, median and mean, respectively. Long boxes and whiskers indicate greater variation. (**a**) salinity stress, (**b**) cold stress, (**c**) drought stress.

The mean Cq values of candidate reference genes in all 18 samples (six samples under each stress condition) ranged from 23.14 (*5.8S rRNA*) to 29.28 (*miR160e*) (Fig. 1). 5.8S *rRNA* was the most highly expressed gene under salinity and cold stresses, and *GADPH* showed the highest level of expression under drought stress, whereas *miR160e* expressed with the lowest level under all three stress conditions. For CV values, the highest and lowest values under the three different treatments were 6.02% (*5.8S rRNA*) and 1.15% (*U6 snRNA*) in salinity stress; 9.73% (*UBQ*) and 0.55% (*U6 snRNA*) in cold stress; and 6.56% (*GAPDH*) and 2.24% (*5.8S rRNA*) in drought stress, respectively (Fig. 1). The rank of mean CV values of candidate reference genes was *U6 snRNA* < *miR168* < *miR160e* < *EF1 < miR164a* < *ACT* < *GAPDH* < *5.8S rRNA* < *UBQ*, which represented the fluctuation difference of Cq values.

### Analysis of gene expression stability

**Figure 2:**
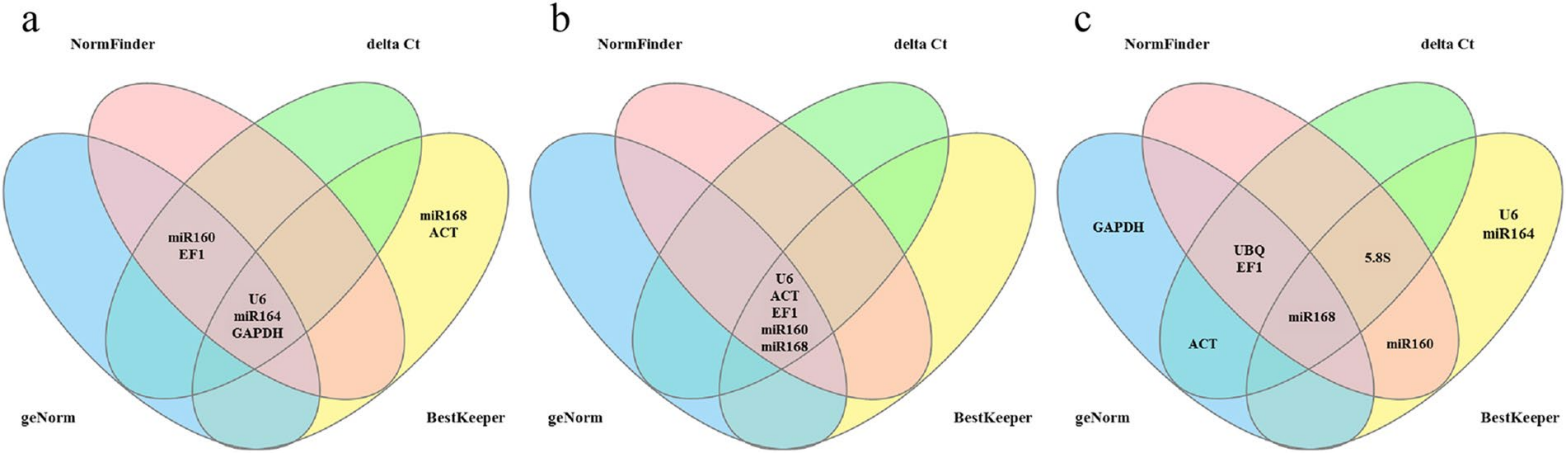
The top 5 most stable reference genes generated by geNorm, NormFinder, deltaCt and Bestkeeper. The blue, pink, green and yellow circles each contain the top 5 most stable reference genes of geNorm, NormFinder, deltaCt and Bestkeeper, respectively. The genes in the overlap area are the ones confirmed as the top 5 most stable reference genes by more than one algorithm. (**a**) salinity stress, (**b**) cold stress, (**c**) drought stress.

Four algorithms including geNorm, NormFinder, deltaCt and Bestkeeper were used to evaluate the expression stability of the candidate reference genes. geNorm is an algorithm of which the core concept is to compare the average pairwise expression ratio between candidate reference genes. The least stable gene can be excluded by a stepwise approach, and then the M value is provided to evaluate a candidate gene. As the algorithm suggests, a gene with an M value over 1.5 is not suitable to be a reference gene. The NormFinder algorithm uses ANOVA-based mathematical analysis to generate the inter- and intra-group variations of candidate genes. For the Bestkeeper algorithm, the lowest coefficient of variance (CV) and standard deviation (SD) are the evaluation criteria for stably expressed reference genes. The principle of the deltaCt algorithm is similar to that of the algorithm of geNorm; the deltaCt method is used to compare the expression stability of pairs of genes. The expression stability values generated from these algorithms were unified as the stability value (SV), and the SVs of candidate reference genes under salinity, cold and drought stress conditions are shown in Table 3–5, respectively. Additionally, the top 5 most stable reference genes generated by each algorithm are presented as the Venn plot in Fig. 2.

**Table 3:** The expression stability of candidate reference genes under salinity stress.

Rank	geNorm		NormFinder		deltaCt		Bestkeeper	
	Gene	SV	Gene	SV	Gene	SV	Gene	SV
1	miR160e	0.216	U6 snRNA	0.012	U6 snRNA	0.617	U6 snRNA	0.208
2	miR164a	0.216	miR164a	0.012	miR164a	0.633	miR164a	0.269
3	U6 snRNA	0.228	EF1	0.014	miR160e	0.685	miR168	0.280
4	GAPDH	0.425	GAPDH	0.015	GAPDH	0.701	GAPDH	0.333
5	EF1	0.475	miR160e	0.016	EF1	0.711	ACT	0.363
6	miR168	0.531	ACT	0.018	ACT	0.732	miR160e	0.370
7	ACT	0.578	miR168	0.023	miR168	0.791	EF1	0.379
8	UBQ	0.620	UBQ	0.024	UBQ	0.842	UBQ	0.410
9	5.8S rRNA	0.788	5.8S rRNA	0.058	5.8S rRNA	1.375	5.8S rRNA	1.142

**Table 4:** The expression stability of candidate reference genes under cold stress.

Rank	geNorm		NormFinder		deltaCt		Bestkeeper	
	Gene	SV	Gene	SV	Gene	SV	Gene	SV
1	miR160e	0.258	U6 snRNA	0.007	U6 snRNA	1.071	U6 snRNA	0.106
2	miR168	0.258	ACT	0.011	miR168	1.169	miR168	0.259
3	U6 snRNA	0.363	miR160e	0.021	ACT	1.208	EF1	0.396
4	EF1	0.593	miR168	0.021	miR160e	1.217	miR160e	0.458
5	ACT	0.709	EF1	0.022	EF1	1.247	ACT	0.692
6	GAPDH	0.824	GAPDH	0.032	GAPDH	1.373	5.8S rRNA	0.796
7	5.8S rRNA	0.939	5.8S rRNA	0.047	5.8S rRNA	1.538	GAPDH	0.867
8	miR164a	1.164	miR164a	0.076	miR164a	2.114	miR164a	1.205
9	UBQ	1.525	UBQ	0.090	UBQ	2.789	UBQ	2.426

**Table 5:** The expression stability of candidate reference genes under drought stress.

Rank	geNorm		NormFinder		deltaCt		Bestkeeper	
	Gene	SV	Gene	SV	Gene	SV	Gene	SV
1	ACT	0.255	miR168	0.024	EF1	1.120	5.8S rRNA	0.427
2	UBQ	0.255	UBQ	0.027	UBQ	1.129	miR168	0.484
3	GAPDH	0.451	EF1	0.027	miR168	1.140	miR164a	0.635
4	EF1	0.519	miR160e	0.030	ACT	1.236	miR160e	0.690
5	miR168	0.845	5.8S rRNA	0.035	5.8S rRNA	1.259	U6 snRNA	0.703
6	miR160e	0.990	ACT	0.036	miR160e	1.260	UBQ	0.914
7	5.8S rRNA	1.104	miR164a	0.042	GAPDH	1.370	EF1	0.952
8	miR164a	1.194	GAPDH	0.050	miR164a	1.427	ACT	1.092
9	U6 snRNA	1.281	U6 snRNA	0.053	U6 snRNA	1.584	GAPDH	1.320

Three stress treatments were conducted in this experiment, and H_2_O_2_ (Hydrogen Peroxide) content, MDA (Malondialdehyde) content and SOD (Superoxide Dismutase) activity were measured to reflect the stress severity (Fig. S2). In salinity-treated samples, *U6 snRNA* and *miR164a* ranked at the top 3 in all algorithms, indicating their stable expression under the condition of salinity stress. However, the least stable of all candidate genes calculated by the four algorithms under salinity stress were *5.8S rRNA* and *UBQ*. For other candidate genes, the rankings were variable among different algorithms. For example, *miR168* was the 6th, 7th, 7th, and 3rd most stable gene evaluated by geNorm, NormFinder, deltaCt and Bestkeeper, respectively (Table 3). Under the cold stress condition, the most stable reference genes were similar across the four algorithms (Table 4), and collectively, *U6 snRNA* and *miR168* were the first and second most stable genes according to three algorithms, whereas *U6 snRNA* and *ACT* were the top two stable genes evaluated by NormFinder. For the least stable genes, the results given by geNorm, NormFinder, deltaCt and Bestkeeper were consistent, which confirmed that *miR164a* and *UBQ* were unstable under cold stress. As noted above, candidate genes with a SV (M) over 1.5 are not suitable to be a reference gene according to the algorithm of geNorm. Among all samples under different treatments, the SVs of 9 candidate genes were all below 1.5, except for *UBQ* in cold-treated samples. *UBQ* had a SV of 1.525 under cold stress, which indicated that *UBQ* was not a suitable reference gene for cold-treated grapevine. For drought stress, the SV rankings of the candidate genes were variable among algorithms (Table 5). However, *UBQ* and *miR168* were indicated as the top 2 most stable genes by at least two algorithms. For the genes with low stability, *U6 snRNA* was the least stable gene from geNorm, NormFinder and deltaCt, whereas *GAPDH* was evaluated as the least stable gene by Bestkeeper. Furthermore, *miR164a* showed an unstable expression trait evaluated by most algorithms under drought stress.

**Figure 3:**
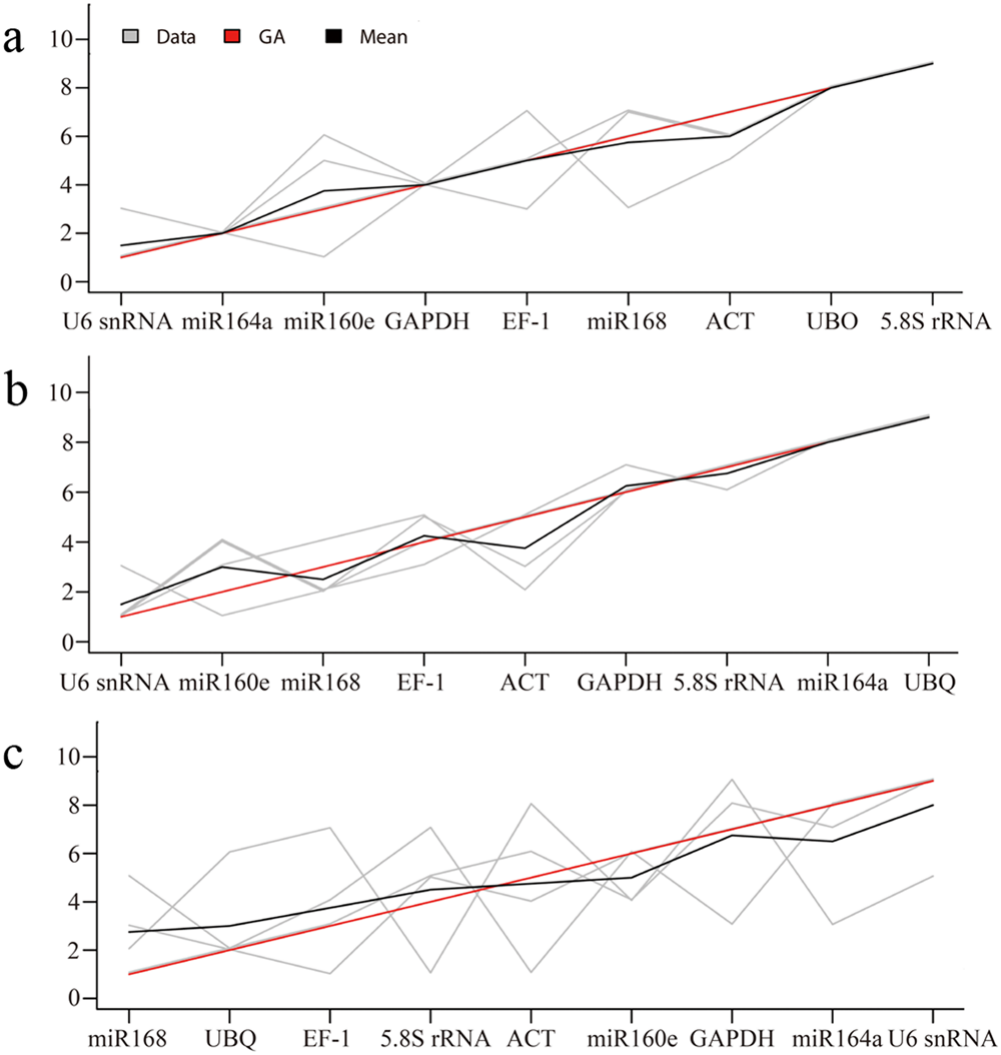
Consensus stability ranking generated by a Monte Carlo algorithm. The R package RankAggreg (Version 0.5) was used to integrate ranking lists of four stability algorithms (geNorm, NormFinder, deltaCt and Bestkeeper) by a Monte Carlo model. Each grey line represents the ranking of an individual algorithm; the black and red lines represent the mean ranking and consensus ranking generated by the Monte Carlo algorithm, respectively. (**a**) salinity stress, (**b)** cold stress, (**c**) drought stress.

Considering the limits and biases of different algorithms, four algorithms were performed to evaluate the expression stability of candidate genes. To reach a consensus on the rankings of optimal reference genes, the results generated by the four algorithms were aggregated by the Cross-Entropy Monte Carlo approach using Rpackage RankAggreg^36^. Based on the aggregated rankings (Fig. 3), the most and least stable candidate genes under different treatments were almost consistent with the results of ranking orders provided by each algorithm (Tables 3–5). *U6 snRNA* and *miR164a*, *U6 snRNA* and *miR160e*, and *miR168* and *UBQ* were the candidate genes ranked at the top 2 under salinity, cold and drought stress, respectively, whereas the least stable candidate genes under these stresses were *UBQ* and *5.8S rRNA*, *miR164a* and *UBQ*, and *miR164a* and *U6 snRNA*, respectively.

### Optimal combinations of reference genes under salinity, cold and drought stresses

**Figure 4:**
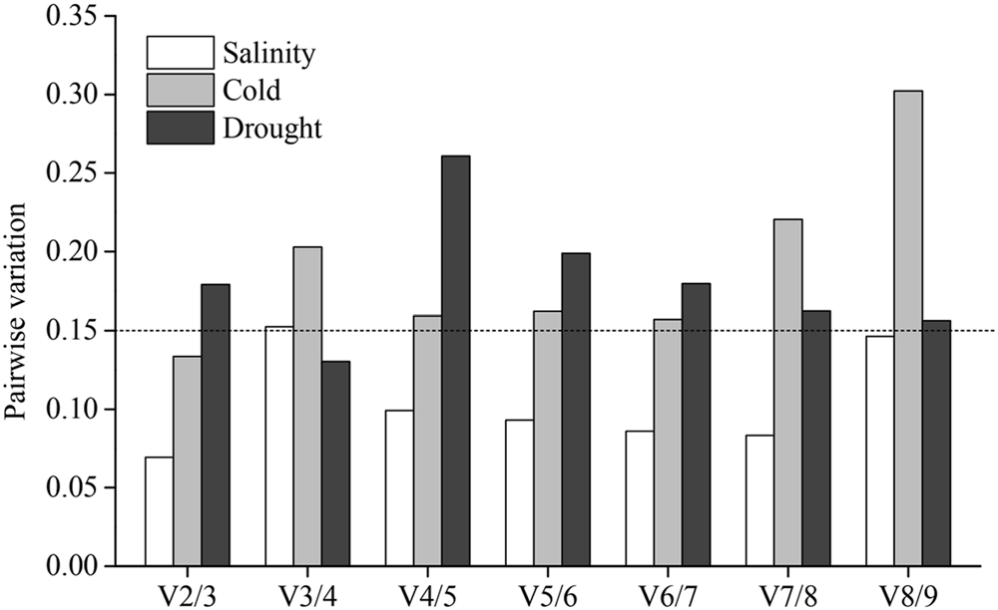
Determination of the optimal number of candidate reference genes by geNorm analysis. Pairwise variation (V_n/n + 1_) between the normalization factors NF_n_ and NF_n+1_ was used to determine the optimal number of multiple reference genes. When the value of V_n/n + 1_ is ≤0.15, the addition of one or more reference genes into the reference gene combination would not be necessary.

As noted above, one reference gene may result in bias during qRT-PCR. Using a reference gene combination can obtain more accurate and reliable normalization of gene expression data compared with that of a single reference gene for qRT-PCR^26^. The optimal number of multiple reference genes was determined by geNorm through calculating the V value (pairwise variation) ratio (V = V_n_/V_n + 1_) between normalization factors (NF). The value of 0.15 was set as the threshold of the V value to determine whether an additional reference gene was required in the reference gene set. When V_n_/V_n + 1_ ≤ 0.15, the n most stable genes were sufficient to create a stable reference gene combination. As shown in Fig. 4, the V_2/3_ of candidate genes under salinity and cold stress was 0.069 and 0.133, respectively, which were below 0.15. Therefore, the optimal reference gene sets under salinity and cold stress were *miR160e* + *miR164a* and *miR160e* + *miR168*, respectively. For drought stress, because V_2/3_ = 0.179 was more than 0.15, whereas V_3/4_ = 0.130 was less than 0.15, the top three candidate genes *ACT*, *UBQ* and *GAPDH* were the best genes to normalize the qRT-PCR results.

### Verification of reference genes

**Figure 5:**
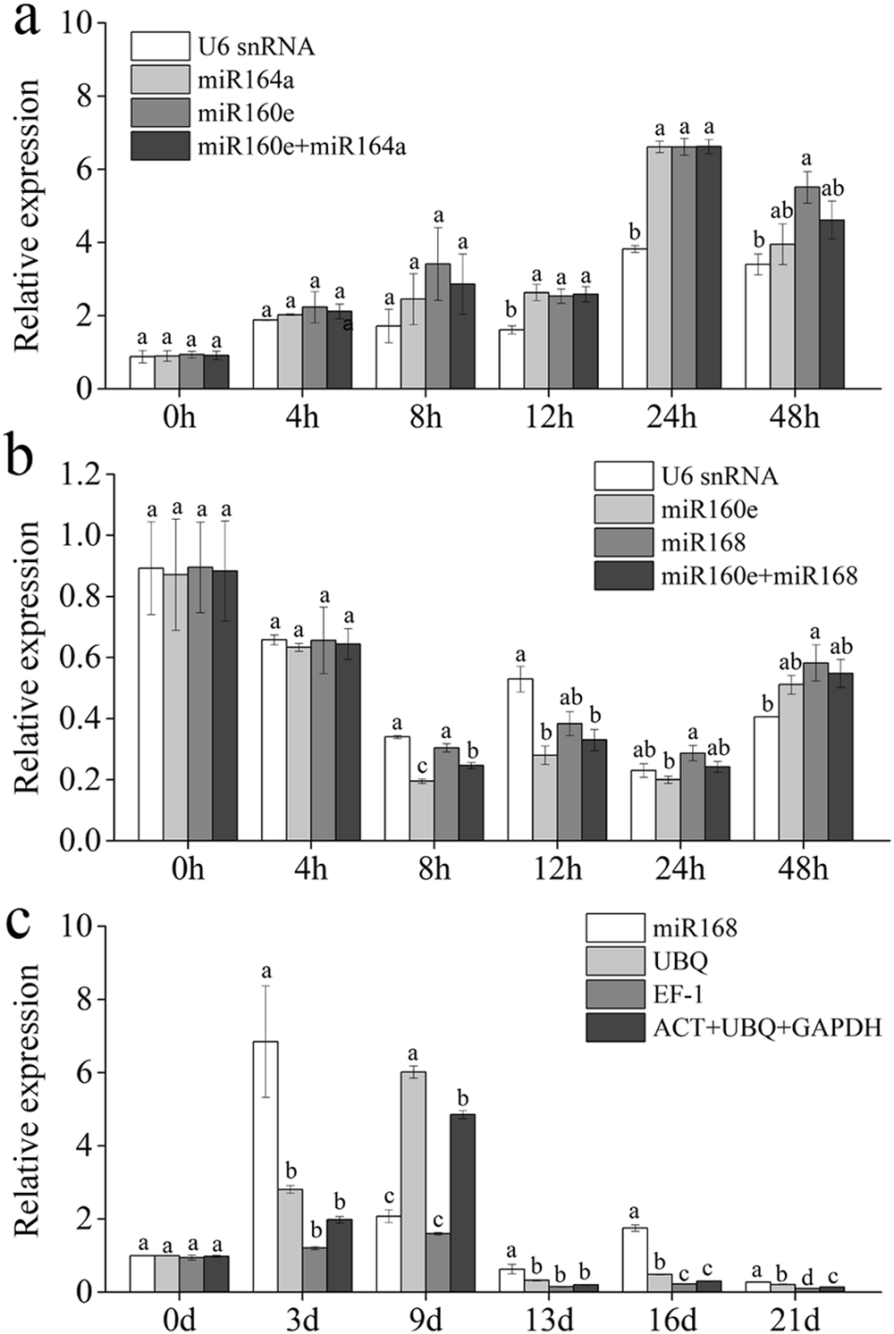
Relative expression of target genes. Different miRNAs were chosen to be target genes for different abiotic stresses. Selected reference genes or gene combination was used to normalize the expression results. (**a**) salinity stress. The columns represent the relative expression of *miR319*. (**b**) cold stress. The columns represent the relative expression of *miR319*. (**c**) drought stress. The columns represent the relative expression of *miR408*.

For each of the three stress conditions, the 3 most stable single reference genes and a gene set were selected to validate the effectiveness of reference genes. Based on the rankings and V values, which were generated by RankAggreg and geNorm, respectively, the reference genes and gene set were selected as follow: *U6 snRNA*, *miR164a*, *miR160e*, and *miR160e* + *miR164a* for salinity stress; *U6 snRNA*, *miR160e*, *miR168*, and *miR160e* + *miR168* for cold stress; *miR168*, *UBQ*, *EF1*, and *ACT* + *UBQ* + *GAPDH* for drought stress. According to previous studies, *miR319* responds to salinity and cold stresses, whereas *miR408* is associated with drought stress in different plant species^37^. Thus, *miR319* and *miR408* were chosen as target genes to perform qRT-PCR to validate the normalization efficiency of selected reference genes. When normalized by *U6 snRNA*, *miR164a*, *miR160e* and *miR160e* + *miR164a*, the expression of *miR319* under salinity stress increased through time until the peak was reached at 24 h, followed by a slight decrease at 48 h (Fig. 5a). The expression patterns normalized by different reference genes or gene set showed consistency. However, at the time points of 12 and 24 h, a significant difference was observed between *U6 snRNA* and the other normalization factors. Under cold stress, *miR319* expression decreased at time points of 8, 12 and 24 h. The expression levels of *miR319* normalized by different reference genes were not exactly the same (Fig. 5b); although the normalization results of *U6 snRNA* and *miR168* were similar, the expression normalized by *miR160e* and *miR160e* + *miR168* showed no significant differences at most time points. For drought stress, the expression patterns of *miR408* with different normalization factors were not as consistent as those under the salinity and cold stresses (Fig. 5c). The expression of *miR408* increased at the time points of 3 and 9 d, followed by a gradual decrease. Significant differences occurred at all time points except for d 0 and were primarily between *miR168* and the other reference genes.

## Discussion

qRT-PCR is a widely used method for gene expression analysis because of the high sensitivity and accuracy^21,38^. However, the final result of qRT-PCR can be easily influenced by technical error because of the advantage of high sensitivity^39^, and such bias may be caused by RNA extraction, cDNA reverse transcription, or the qRT-PCR process^27^. To obtain a more reliable result, the most common approach is to introduce one or more reference genes as the normalization factors^25,40^. According to previous research, the most suitable reference genes for a given experimental condition, such as a biotic or abiotic stress, may be different from the suggested reference genes for other conditions^41,42^. The optimal reference genes should be validated for different species, treatments or specific tissues.

Several studies have conducted reference gene validation experiments for grapevine mRNA qRT-PCR but not for grapevine miRNA qRT-PCR^43,44^. In these studies, protein-coding genes (such as *ACT* and *UBQ*) or ribosome RNAs (such as *5.8S rRNA* and *18S rRNA*) are always considered the most appropriate normalization factors for mRNA quantification. However, whether these selected reference genes are also suitable for grapevine miRNA qRT-PCR remains unclear. Because of the lack of supporting evidence, the widely used reference genes in other species, such as *U6 snRNA* and *5.8S rRNA*, are usually chosen for normalizing grapevine miRNA quantitation data^28^. In addition to mRNAs, rRNAs and snRNAs, the potential of miRNAs as reference genes has received much attention recently. In some plant species, such as lettuce^31^, wheat^29^ and peach^32^, miRNAs are more stable than the currently used reference genes under specific conditions. Furthermore, compared with other types of RNAs, miRNA is much shorter in length, and therefore, miRNA extraction and reverse transcription are different from the protocols for longer RNAs^28^. Using miRNAs as reference genes could avoid the bias caused by different RNA extraction and reverse transcription processes between reference genes and target genes^45^. Therefore, to guarantee a comprehensive comparison, the candidate reference genes of this research included traditional housekeeping genes (*ACT*, *UBQ*, *GAPDH* and *EF1*), widely used reference genes in miRNA qRT-PCR (*5.8S rRNA* and *U6 snRNA*), and conserved miRNAs (*miR160e*, *miR164a* and *miR168*). The genes *miR156a*, *miR159a*, *miR162*, *miR167a*, *miR169a*, and *miR396a* were excluded from the candidate list because of unsatisfactory amplification efficiency or regression coefficient of the standard curve.

Because of no consensus about the most reliable gene expression stability evaluation algorithm, four algorithms (geNorm, NormFinder, deltaCt, Bestkeeper) were used to minimize the bias^46^. *U6 snRNA* was the most stable reference gene under salinity and cold stress in this research, which ranked first in NormFinder, deltaCt, Bestkeeper and in the comprehensive ranking. *U6 snRNA* is one of the most commonly used reference genes in miRNA qRT-PCR^27^ and is reported as the most stable gene in reference gene selection research of citrus^45^ and tea^47^. According to previous research, *U6 snRNA* is highly conserved among species^48^ and functions as a primary component of the RNA spliceosome, participating in the process of mRNA precursors^49,50^. However, *U6 snRNA* ranked at the bottom under drought stress, as evaluated by geNorm, NormFinder and deltaCt, which could be because drought stress is lethal for plants^5^. Under drought stress, maintaining normal physiology processes in leaves is difficult, which leads to the decrease of mRNA content and that of *U6 snRNA* in the late stage of drought stress^51^. Another traditional miRNA reference gene *5.8S rRNA* was less stable than *U6 snRNA* under salinity and cold stresses but was more stable than *U6 snRNA* under drought stress. This result indicates that no reference gene is suitable for every experimental condition. The test of expression stability under a particular experimental condition is always required, when not previously confirmed.

*miR160e*, *miR164a* and *miR168* were ranked at the top one or top two under cold, salinity and drought stresses, indicating these genes were more stable than protein-coding genes and *5.8S rRNA*. This result is consistent with previous studies^29,31,32^, indicating miRNAs are qualified to be miRNA qRT-PCR reference genes for grapevine under abiotic stresses. These 3 miRNAs all belong to conserved miRNA families and undertake important physiological functions. The target genes of *miR160e* are *ARF* family members that respond to auxin and associate with the development of multiple organs, such as seeds, leaves, roots and flowers^52^. For *miR168*, the function is regulation of the ARGONAUTE1 protein^53^, which is the primary component of the RNA-induced slicing complex (RISC) and influences plant growth and development^54,55^. Because of the involvement in fundamental physiological processes, the stable expression of *miR160e* and *miR168* was a reasonable result. *miR164a* reportedly negatively regulates the expression of *NAC*, a transcription factor that responds to salinity stress. However, in this research, *miR164a* showed relatively high stability under salinity stress, which is consistent with a previous report in maize^56^. In the maize experiment, *NAC* showed a more dramatic response to salinity stress after treatment, whereas *miR164a* only changed moderately, decreasing to approximately 90% relative expression on average compared with that of the control.

As reported previously, using a reference gene set instead of a single reference gene can provide more reliable normalized results^25,26^. According to geNorm, *miR160e* + *miR164a*, *miR160e* + *miR168*, and *ACT* + *UBQ* + *GAPDH* were selected as the most suitable reference gene sets for salinity, cold and drought stresses, respectively. To validate the selected reference genes or gene sets, the top 3 genes of comprehensive ranking under each experimental treatment with the gene sets mentioned above were used to normalize the expression of target genes. Note that the genes with a low or middle ranking also have the potential to be reference genes as long as their SVs meet the requirements of algorithms in some other stress conditions^42^. As shown in Fig. 5, under salinity and cold stresses, the quantification results of target gene *miR319* exhibited similar patterns when normalized by the selected reference genes or gene sets, demonstrating that the tested reference gene or gene set was mutually validated. For drought stress, when normalized by *miR168* and the others, the expression of target gene *miR408* showed disagreement. The phenomenon that different reference genes lead to different expression patterns is also observed in other research, further indicating the importance of selecting the proper reference gene^31,57^.

In conclusion, in grapevine, *U6 snRNA*, *miR164a*, *miR160e* and *miR160e* + *miR164a* are all suitable reference genes or gene set under salinity stress, whereas *U6 snRNA*, *miR160e*, *miR168* and *miR160e* + *miR168* can be used for qRT-PCR normalization under cold stress. Considering the target gene was miRNA, and *miR168* was the top 1 reference gene of comprehensive ranking under drought stress, we suggest *miR168* as the reference gene for miRNA qRT-PCR, rather than the others.

## Methods

### Plant materials and treatments

This study was performed on one-year-old *Vitis vinifera* L. cv. Muscat Hamburg grown in the Fruit Experimental Greenhouse of Shanghai Jiao Tong University. These self-rooted grapevines were potted into a soil mixture (garden soil: organic fertilizer: perlite = 1: 1: 1) in 15 L pots. Fifty-four plants were divided equally into three groups to conduct stress treatments after cultivating under greenhouse conditions for 10 weeks. For salinity stress, plants were treated with 200 mM NaCl water solution for several days (sampling times are listed below); for cold stress, plants were exposed to low temperature in an incubator (4 °C, 16 h light/8 h dark); for drought stress, plants were maintained with the water supply withheld. Leaves were collected at 0, 4, 8, 12, 24, and 48 h after salinity and cold treatments and at 0, 3, 9, 13, 16, and 21 d after drought treatment. Three biological replications were included at each time point. All samples were immediately frozen in liquid nitrogen and stored at −80 °C until used. H_2_O_2_ content, MDA content and SOD activity were measured using Hydrogen Peroxide assay kit (Nanjing Jiancheng, Nanjing, Jiangsu, China), Malondialdehyde assay kit (Nanjing Jiancheng, Nanjing, Jiangsu, China) and Total Superoxide Dismutase assay kit (Nanjing Jiancheng, Nanjing, Jiangsu, China), respectively.

### RNA extraction and cDNA synthesis

Total RNA was isolated using RNAplant Plus Reagent (TIANGEN, Beijing, China), and genomic DNA was removed using Recombinant DNase I (TAKARA Biotechnology, Dalian, Liaoning, China). RNA integrity was evaluated by 2% agarose gel electrophoresis; whereas a NanoDrop 2000 measured RNA concentration and quality (Thermo Scientific, Waltham, Massachusetts, USA). After analysis, samples with an OD 260/280 ratio from 1.9 to 2.1 and an OD 260/230 ratio from 1.8 to 2.2 were chosen for the next step.

The first-strand cDNA was synthesized from 1 μg of total RNA according to the instructions of a FastKing RT Kit (TIANGEN, Beijing, China). The 20 μL reaction system was performed at 42 °C for 15 min and 95 °C for 3 min. For miRNA amplification, cDNA synthesis was conducted using the same protocol described above, except stem-loop reverse transcription primers were used instead of oligo (dT).

### Selection of reference genes and primer design

A total of 15 candidate reference genes were selected. The sequences of *5.8S rRNA* and *U6 snRNA*, the two most commonly used reference genes for miRNA qRT-PCR, were obtained from NCBI (GenBank Accession: KT344661.1) and reported by Kullan *et al*.^58^, respectively. The sequences of 4 traditional housekeeping genes, *ACT*, *UBQ*, *GAPDH* and *EF1*, were reported by Reid *et al*.^59^. According to the miRNA high-throughput sequencing results in grapevine^28,60^ and reference gene selection research for miRNA qRT-PCR in other plant species^27,29,32^, *miR156a*, *miR159a, miR160e*, *miR162*, *miR164a*, *miR167a*, *miR168*, *miR169a*, and *miR396a* were chosen as potential normalization factors for miRNA qRT-PCR in grapevine.

The primers of *5.8S rRNA*, *ACT*, *UBQ*, *GAPDH* and *EF1* were designed by Primer Express (3.0.1), whereas the primers of *U6 snRNA* were obtained from Kullan *et al*.^58^. For miRNA cDNA synthesis, the stem-loop primers were designed according to Chen^21^, which consisted of 44 conserved and 8 variable nucleotides that were specific to the 3′ end of the miRNA sequences. For miRNA qRT-PCR, the forward primers were designed using 1–11th nucleotides of miRNA sequences, with 7 nucleotides (GCGGCGG) added to the 5′ end to increase the length of primers; whereas the reverse primers were universal.

### qRT-PCR analysis

qRT-PCR was performed on a LightCycler480 System (Roche, New York, USA). The 10 μL reaction system contained 5 μL of SYBR® Premix Ex Taq (TAKARA Biotechnology, Dalian, China), 0.3 μM each primer, 2 μL of diluted cDNA (miRNA cDNA and other cDNA diluted 25-fold and 50-fold, respectively) and 2.4 μL of ddH_2_O. The procedure was conducted as follows: 95 °C for 10 min, amplification for 40 cycles (95 °C for 20 s, 60 °C for 20 s, 75 °C for 20 s). Negative controls were set to test for possible contamination, and the melting curves were evaluated in each reaction to ensure the specificity of amplified action. Each sample was evaluated in three technical triplicates.

Using a series of 5-fold diluted cDNA as templates, the standard curves were generated for each candidate reference gene. The correlation coefficient (R^2^) and slope were obtained from the linear regression model created by the LightCycle 480 system, and the PCR efficiency (E) was calculated using the following formula:

$$E={10}^{-1/slope}-1$$

### Data analyses

The expression stability of candidate reference genes was calculated using geNorm, NormFinder, deltaCt and Bestkeeper. As the algorithms required, Cq values of all samples (six time points under salinity, cold and drought stress) were transformed into Q values using the formula *Q* = *E*^Δ*Cq*^. ΔCq indicates the difference between the sample Cq and the lowest Cq among the samples of six time points. Bestkeeper and deltaCt were performed by the online analysis tool RefFinder (http://fulxie.0fees.us/?type=reference). To integrate the ranks of the four algorithms, RankAggreg Rpackage (Version 0.5) was used to generate a consensus ranking of candidate reference genes.

### Reference gene validation

As reported by Thiebaut *et al*.^61^, Zhou *et al*.^62^ and Ma *et al*.^37^, *miR319* is responsive to salt and cold stress, whereas *miR408* is responsive to drought stress. Therefore, *miR319* (salinity and cold stress) and *miR408* (drought stress) were chosen as the target genes to validate the stability of selected reference genes. The relative expression of the target genes at six time points was calculated using the 2^−ΔΔCt^ method, and the significant difference analysis was performed using the SPSS statistical software package (19.0).

## Electronic supplementary material

Figure S1 and Figure S2
