# Supplementary material for: Selection of reference genes for miRNA qRT-PCR under abiotic stress in grapevine
Source: Sci Rep. 2018 Mar 13;8:4444. doi: 10.1038/s41598-018-22743-6 (PMC5849727; doi:10.1038/s41598-018-22743-6)
Supplement: Supplementary file 1 — Figure S1 and Figure S2 [file 41598_2018_22743_MOESM1_ESM.pdf]

Selection of reference genes for miRNA qRT-PCR under abiotic stress in grapevine

Meng Luo[1, 2], Zhen Gao[1, 2], Hui Li[1, 2], Qin Li[1, 2], Caixi Zhang[1, 2], Wenping Xu[1, 2], Shiren Song[1, 2], Chao Ma[1, 2]*, Shiping Wang[1, 2, 3]*

[1]Department of Plant Science, School of Agriculture and Biology, Shanghai Jiao Tong University, Shanghai, China

[2]Center for Viticulture and Enology, School of Agriculture and Biology, Shanghai Jiao Tong University, Shanghai 200240, China.

[3]Institute of Agro-food Science and Technology, Key Laboratory of Agro-products Processing Technology of Shandong, Shandong Academy of Agricultural Sciences, Jinan 250100, People's Republic of China

*** Corresponding author:** Department of Plant Science, School of Agriculture and Biology, Shanghai Jiao Tong University, Shanghai, China.

Tel& Fax: +0086-21-34205961

E-mail address: chaoma2015@sjtu.edu.cn, fruit@sjtu.edu.cn

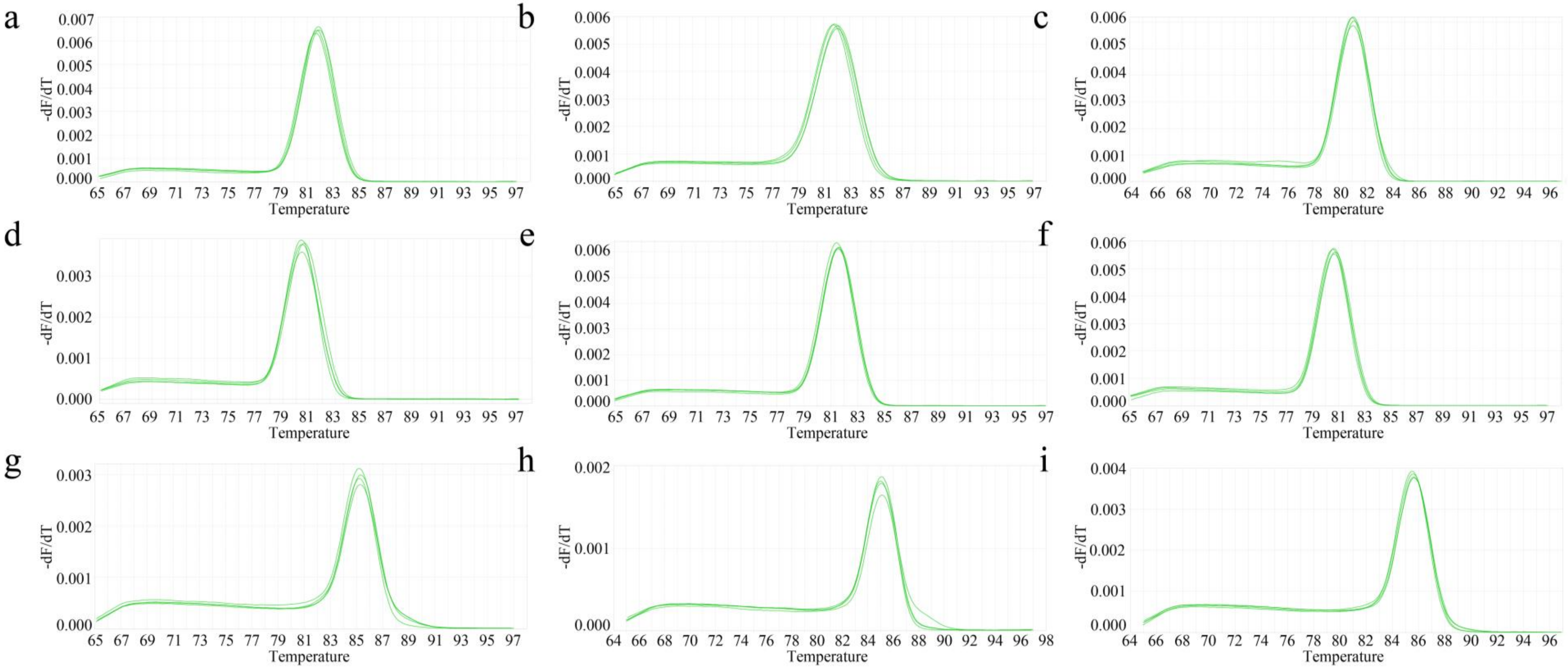

Supplementary Figure S1. The melting peaks of candidate reference genes. Plots a-i represent the melting peaks of *5.8S rRNA*, *U6 snRNA*, *ACT, UBQ, GAPDH, EF1*, *miR160e*, *miR164a*, and *miR168*, respectively.

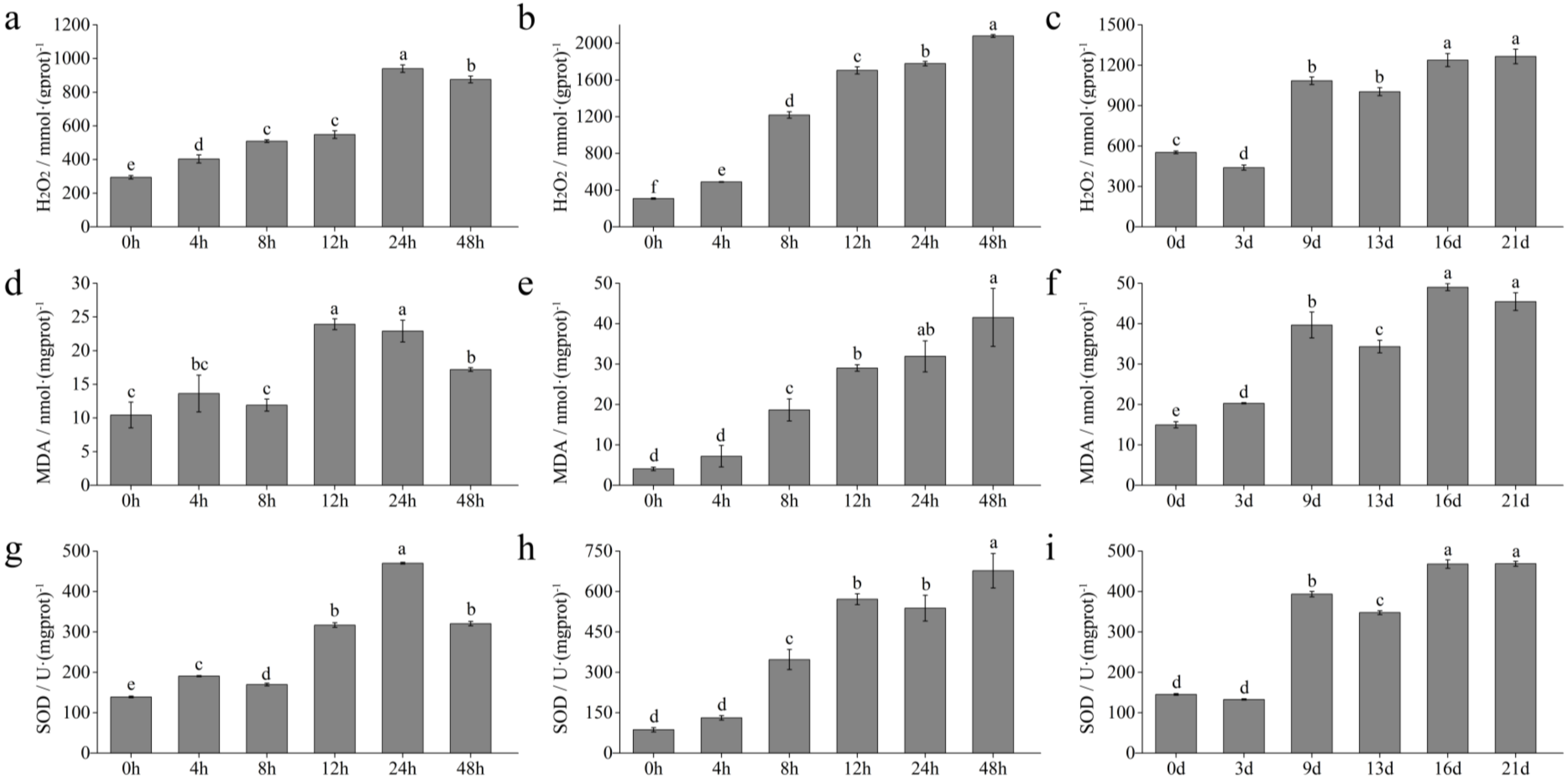

Supplementary Figure S2. The physiological response of grapevine leaves for different stresses. Plots a-c represent the H_2O_2 content at six sampling time points under salinity, cold and drought stress, respectively; Plots d-f represent the MDA content at six sampling time points under salinity, cold and drought stress, respectively; Plots g-i represent the SOD activity at six sampling time points under salinity, cold and drought stress, respectively.
